# HIV-1 Protease, Reverse Transcriptase, and Integrase Variation

**DOI:** 10.1128/JVI.00495-16

**Published:** 2016-06-10

**Authors:** Soo-Yon Rhee, Kris Sankaran, Vici Varghese, Mark A. Winters, Christopher B. Hurt, Joseph J. Eron, Neil Parkin, Susan P. Holmes, Mark Holodniy, Robert W. Shafer

**Affiliations:** a Department of Medicine, Stanford University, Stanford, California, USA; b Department of Statistics, Stanford University, Stanford, California, USA; c Office of Public Health, Department of Veterans Affairs, Washington, DC, USA; d Institute for Global Health and Infectious Diseases, University of North Carolina at Chapel Hill, Chapel Hill, North Carolina, USA; e Data First Consulting, Belmont, California, USA; Institute of Molecular Virology

## Abstract

HIV-1 protease (PR), reverse transcriptase (RT), and integrase (IN) variability presents a challenge to laboratories performing genotypic resistance testing. This challenge will grow with increased sequencing of samples enriched for proviral DNA such as dried blood spots and increased use of next-generation sequencing (NGS) to detect low-abundance HIV-1 variants. We analyzed PR and RT sequences from >100,000 individuals and IN sequences from >10,000 individuals to characterize variation at each amino acid position, identify mutations indicating APOBEC-mediated G-to-A editing, and identify mutations resulting from selective drug pressure. Forty-seven percent of PR, 37% of RT, and 34% of IN positions had one or more amino acid variants with a prevalence of ≥1%. Seventy percent of PR, 60% of RT, and 60% of IN positions had one or more variants with a prevalence of ≥0.1%. Overall 201 PR, 636 RT, and 346 IN variants had a prevalence of ≥0.1%. The median intersubtype prevalence ratios were 2.9-, 2.1-, and 1.9-fold for these PR, RT, and IN variants, respectively. Only 5.0% of PR, 3.7% of RT, and 2.0% of IN variants had a median intersubtype prevalence ratio of ≥10-fold. Variants at lower prevalences were more likely to differ biochemically and to be part of an electrophoretic mixture compared to high-prevalence variants. There were 209 mutations indicative of APOBEC-mediated G-to-A editing and 326 mutations nonpolymorphic treatment selected. Identification of viruses with a high number of APOBEC-associated mutations will facilitate the quality control of dried blood spot sequencing. Identifying sequences with a high proportion of rare mutations will facilitate the quality control of NGS.

**IMPORTANCE** Most antiretroviral drugs target three HIV-1 proteins: PR, RT, and IN. These proteins are highly variable: many different amino acids can be present at the same position in viruses from different individuals. Some of the amino acid variants cause drug resistance and occur mainly in individuals receiving antiretroviral drugs. Some variants result from a human cellular defense mechanism called APOBEC-mediated hypermutation. Many variants result from naturally occurring mutation. Some variants may represent technical artifacts. We studied PR and RT sequences from >100,000 individuals and IN sequences from >10,000 individuals to quantify variation at each amino acid position in these three HIV-1 proteins. We performed analyses to determine which amino acid variants resulted from antiretroviral drug selection pressure, APOBEC-mediated editing, and naturally occurring variation. Our results provide information essential to clinical, research, and public health laboratories performing genotypic resistance testing by sequencing HIV-1 PR, RT, and IN.

## INTRODUCTION

As HIV-1 has spread among humans, it has developed an extraordinary amount of genetic diversity (1). This diversity arises from HIV-1's high mutation rate and predilection for recombination (2, 3). Amino acid variants accumulate within an individual as a result of various selective pressures and HIV-1's genetic robustness or tolerance for a large number of different amino acid variants (4, 5). The large number of protease (PR), reverse transcriptase (RT), and integrase (IN) amino acid variants has implications for antiretroviral (ARV) therapy and presents a challenge to laboratories performing genotypic resistance testing.

The challenge of HIV-1 genotypic resistance test interpretation is increasing with the adoption of dried blood spot sequencing in low- and middle-income countries and the expansion of next-generation sequencing (NGS) in upper-income countries. Dried blood spot samples contain proviral DNA, which is more likely to contain APOBEC-mediated G-to-A hypermutation, an ancient host defense mechanism responsible for lethal mutagenesis (6). NGS technologies are intrinsically more error prone than dideoxynucleotide terminator Sanger sequencing and are at risk of yielding reports of low-abundance variants that result from PCR error (7, 8).

We analyzed PR and RT direct PCR Sanger sequences from more than 100,000 individuals and IN direct PCR Sanger sequences from more than 10,000 individuals to characterize the amino acid variation at each amino acid position in these genes. We also analyzed sequences from individuals with known ARV treatment histories to identify those mutations resulting from selective drug pressure. Knowledge of the observed variation and selection pressure on the molecular targets of HIV therapy can be useful to clinical, research, and public health laboratories performing genotypic resistance testing.

## MATERIALS AND METHODS

### Sequences.

HIV-1 group M protease (PR), reverse transcriptase (RT), and integrase (IN) sequences determined by direct PCR dideoxynucleotide sequencing were retrieved from the Stanford HIV Drug Resistance Database (HIVDB) on 1 April 2015 (9). These sequences included 119,000 PR, 128,000 RT, and 13,000 IN sequences from 132,000 individuals in 143 countries. Eighty-five percent of the sequences are in GenBank; 15% were submitted directly to HIVDB. The subtype of each sequence was determined using the REGA HIV-1 Subtyping Tool version 3 (10). The five most common subtypes were B (61%), C (12%), CRF01_AE (8%), CRF02_AG (5%), and A (5%). Clonal sequences were excluded to minimize the likelihood of detecting random virus polymerization errors or—in the case of molecular cloning—PCR errors (11).

Ninety-four percent of sequences were obtained from plasma. Plasma sequences were used to analyze overall amino acid variation and ARV selection pressure. Six percent of sequences were obtained from peripheral blood mononuclear cell (PBMC) proviral DNA. PBMC sequences were pooled with the plasma virus sequences in our analysis of APOBEC-associated mutations because proviral DNA is enriched for APOBEC-edited virus genomes (12, 13).

### APOBEC-associated mutations.

To identify amino acid changes consistent with APOBEC editing, we first identified all highly conserved GG or GA dinucleotide positions in PR, RT, and IN sequences from plasma samples. Conserved dinucleotides were defined as those present in 98% of pooled samples and in each of the five most common subtypes. We then identified sequences containing mutations that resulted from canonical APOBEC3G (GG→AG) and 3F (GA→AA) G-to-A changes at these highly conserved dinucleotide positions. Sequences with these candidate APOBEC-associated mutations were then examined for stop codons—a specific indicator of APOBEC-mediated editing of tryptophan codons (TGG)—and for the number of additional candidate APOBEC-associated mutations.

To identify the number of APOBEC-associated mutations to use as a cutoff for classifying a sequence as likely to have undergone G-to-A hypermutation, we assumed a mixture of two Poisson distributions with different λ's defined as the average number of APOBEC-associated mutations in a sequence: (i) a distribution with a lower λ reflecting sequences lacking APOBEC-associated mutations or containing sparse APOBEC-associated mutations resulting from random HIV mutations and (ii) another distribution with a higher λ reflecting sequences with abundant APOBEC-associated mutations resulting from host APOBEC-3F and APOBEC-3G enzymatic activity. We then developed an R package, LocFDRPois, to estimate the local false discovery rate for each number of APOBEC-associated mutations at which a sequence with that number of APOBEC-associated mutations did not arise from APOBEC editing (http://cran.r-project.org/web/packages/LocFDRPois/).

Theoretically APOBEC-edited genomes should not be found in plasma at a detectable level by Sanger sequencing because these viruses usually cannot complete a virus replication cycle (14). However, plasma can occasionally be contaminated by proviral DNA, which would be extracted and amplified by most HIV sequencing protocols. Therefore, in our subsequent analyses, we excluded all sequences likely to be hypermutated.

### Amino acid variants.

To characterize variability at each position in PR, RT, and IN, we determined the proportion of each amino acid at each position in all viruses and in each of the five most common HIV-1 subtypes. Each amino acid variant was also characterized by its biochemical relatedness to the consensus amino acid at that position using the BLOSUM62 and BLOSUM80 amino acid similarity matrices. The BLOSUM62 and BLOSUM80 matrices are based on the likelihood that two amino acids can replace one another in genomes that share up to 62% and 80% amino acid similarity, respectively, regardless of the organisms from which they were obtained. Thus, they represent the extent of biochemical similarity between amino acids, which is independent of historical evolution and local sequence context. For notational purposes, amino acid variants were defined as differences from the consensus subtype B amino acid sequence because this is a commonly used reference and because it was nearly always the same as the consensus of all pooled sequences.

We also determined the proportion of times that each amino acid variant occurred as part of an electrophoretic mixture in which two peaks were present on the sequence electropherogram resulting in one of the following ambiguous nucleotide calls: R (combination of A and G), Y (combination of C and T), M (combination of A and C), W (combination of A and T), K (combination of G and T), and S (combination of C and G) (15). Amino acids that always occurred as part of an electrophoretic mixture were excluded.

### Nonpolymorphic TSMs.

To identify nonpolymorphic treatment-selected mutations (TSMs), we examined the treatment history of the individuals from whom each sequenced virus was obtained. For each drug class—PR inhibitor (PI), nucleoside RT inhibitor (NRTI), nonnucleoside RT inhibitor (NNRTI), and IN strand transfer inhibitor (INSTI)—sequences were characterized as being either from an ARV class-naive individual who received no drugs belonging to the class or an ARV class-experienced individual who received at least one drug from that class. Sequences from individuals of unknown or uncertain treatment history were excluded from this analysis. In sequences from patients with multiple virus isolates, mutations occurring in more than one isolate were counted just once.

We then examined each amino acid variant for its association with ARV selection pressure. The proportion of each variant in ARV-experienced individuals was compared to its proportion in ARV-naive individuals using a chi-square test with Yates' correction. The Holm's method was then used to control the family-wise error rate for multiple-hypothesis testing at an adjusted *P* value of <0.01 (16). To exclude TSMs under minimal drug selection pressure, we included only those TSMs that were five times more frequent in ARV-experienced than in ARV-naive individuals. To identify the TSMs that are most specific for ARV selection across subtypes, we identified those TSMs that were nonpolymorphic in the absence of selective drug pressure, defined as occurring at a frequency below 1.0% in ARV-naive individuals infected with viruses belonging to each of the five most common subtypes.

Transmitted drug resistance (TDR) will cause many nonpolymorphic TSMs to appear in virus sequences from untreated individuals. This will cause the proportion of these mutations in ARV-naive individuals to be higher than what would be expected in ARV-naive individuals whose viruses had not experienced selective drug pressure. This in turn will reduce the ratio of the prevalence of these mutations in ARV-experienced individuals divided by their inflated prevalence in ARV-naive individuals. Therefore, we restricted our analysis of ARV-naive sequences to those lacking any of the 93 surveillance drug resistance mutations (SDRMs) that have become established markers of TDR (17). For IN for which the SDRM list is not available, we used major INSTI resistance mutations defined in Stanford HIVDB: T66I/A/K, E92Q, F121Y, G140S/A/C, Y143C/R/H, S147G, Q148H/K/R, and N155H/S.

Among RT inhibitor (RTI)-experienced individuals, 75% received NRTIs in combination with an NNRTI, 22% received NRTIs without an NNRTI, and 3% received an NNRTI without an NRTI. The frequent use of NRTIs in combination with an NNRTI makes it difficult to determine for some mutations whether they are selected by NRTIs or NNRTIs. Therefore, we first determined whether RT mutations were treatment selected by comparing the proportions of mutations in sequences from RTI-naive and RTI-experienced individuals. We then determined whether the selection appeared to be primarily associated with NRTIs versus NNRTIs using a previously described approach (18). Those mutations that did not demonstrate a strong significant association with just one class were classified as (i) NRTI associated if their positions are known to be associated with NRTI resistance, (ii) NNRTI associated if their positions are known to be associated with NNRTI resistance, or (iii) undifferentiated RTI associated if their positions were not previously associated with NRTI or NNRTI resistance.

### Synonymous and nonsynonymous mutation rates.

To determine whether the overall nucleotide mutation rate at a codon influenced the likelihood of developing amino acid variants, we estimated the synonymous and nonsynonymous rates at each codon in PR, RT, and IN for the five most common subtypes. For each subtype, we used FastML (19) to determine the most probable ancestral codon and then compared the codon of each sequence to this codon to estimate the number of synonymous changes/number of potential synonymous changes (*dS*) and the number of nonsynonymous changes/number of potential nonsynonymous changes (*dN*). Additionally, we examined each consensus amino acid and TSM to determine the minimum number of nucleotide differences between their respective codons.

## RESULTS

### Signature mutations indicating APOBEC-mediated editing.

Of 297 PR nucleic acids, 24 GG and GA dinucleotides at 22 amino acid positions were conserved in more than 98% of sequences in each of the most common five subtypes. Canonical APOBEC-mediated changes at these positions—GG→AG, GA→AA, and GG→AA (if GG is followed by G)—would result in 58 different amino acid mutations and two stop codons. Fifty of the 58 mutations occurred in sequences from one or more plasma samples. Of the 50 observed mutations, 32 were strongly associated with one or more stop codon or with a canonical APOBEC-mediated mutation at one or more of the active-site residues D25, G27, G49, G51, and G52. Table S1 in the supplemental material lists the two stop codons and the 32 PR mutations, which our analysis suggests indicate APOBEC-mediated editing.

Of 1,680 RT nucleic acids, 128 GG and GA dinucleotides at 115 amino acid positions were conserved in >98% of sequences in each of the five most common subtypes. Canonical APOBEC-mediated changes at these positions would result in 241 different amino acid mutations and 19 stop codons. One hundred eighty of the 245 mutations occurred in sequences from one or more plasma samples. Of the 180 observed mutations, 89 were significantly associated with one or more of stop codons or with a canonical APOBEC-mediated mutation at one of the active-site residues D110, D185, and D186. One of the 89 mutations, M230I, has recently been reported to cause resistance to the NNRTI rilpivirine (20). Table S1 in the supplemental material lists the 19 stop codons and the 88 RT mutations that our analysis suggests indicate APOBEC-mediated editing.

Of the 864 IN nucleic acids, 76 GG and GA dinucleotides at 65 amino acid positions were conserved in >98% of sequences in each of the five most common subtypes. Canonical APOBEC-mediated changes at these positions would result in 136 different amino acid mutations and 7 stop codons. Eighty of the 136 mutations occurred in sequences from one or more plasma samples. Of these 80 mutations, 62 were significantly associated with one or more stop codons or with a canonical APOBEC-mediated mutation at one of the active-site residues D64, D116, and E152. One of the 62 mutations, G118R, has recently been reported to reduce susceptibility to multiple INSTIs (21, 22). Table S1 in the supplemental material lists the seven stop codons and the 61 IN mutations that our analysis suggests indicate APOBEC-mediated editing.

The local false discovery rate derived from the mixture model described in Materials and Methods was used to classify sequences as hypermutated or nonhypermutated based on the number of signature APOBEC mutations within PR, RT, and IN (see Table S2 in the supplemental material). The presence of one signature mutation predicted risks of hypermutation of 18%, 19%, and 16% for PR, RT, and IN sequences, respectively. The presence of two signature mutations predicted risks of hypermutation of 86%, 79%, and 76%, respectively. The presence of three signature mutations predicted risks of hypermutation of 99.8%, 98.5%, and 97.8%, respectively. Therefore, in our subsequent analyses, we excluded 112 PR, 225 RT, and 81 IN plasma sequences containing two or more signature APOBEC mutations.

### Amino acid variation.

Overall, we analyzed 110,357 PR sequences obtained from 101,154 individuals, 118,246 RT sequences from 108,681 individuals, and 11,838 IN sequences from 11,156 individuals. Most RT sequences did not encompass the 3′ RNase H coding region of RT. Therefore, for our analysis of RT amino acid variability, we included just positions 1 to 400.

**FIG 1:**
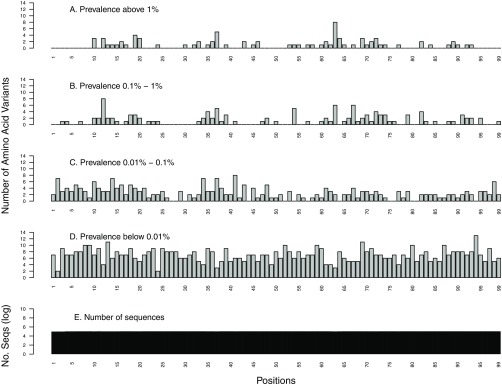
Distribution of the number of HIV-1 protease (PR) amino acid variants by position stratified by prevalence: ≥1% (A), 0.1% to 1% (B), 0.01% to 0.1% (C), and <0.01% (D). The total number of sequences analyzed at each position is shown on a log_10_ scale (E).

Of the 99 PR positions, 47 (47%) had one or more variants occurring at a prevalence of ≥1%, and 69 (70%) had one or more variants occurring at a prevalence of ≥0.1% (Fig. 1). Overall, there were 201 variants occurring at a prevalence of ≥0.1% at these 69 positions (Table 1).

**TABLE 1:** Amino acid variants according to frequency^*a*^

Frequency (%)	Protease				Reverse transcriptase				Integrase			
	No. of amino acid variants	% of positions with variant	Median similarity score^*b*^	% found in electrophoretic mixtures	No. of amino acid variants	% of positions with variant	Median similarity score^*b*^	% found in electrophoretic mixtures	No. of amino acid variants	% of positions with variant	Median similarity score^*b*^	% found in electrophoretic mixtures
<0.01	655	100	−2	60	2,487	99	−2	60	504	85	−1	54
0.01–0.1	260	89	−1	45	1,091	91	−1	49	460	81	0	45
0.1–1	119	56	0	26	379	47	0	30	214	47	0	26
1–10	65	38	0	17	202	31	1	18	107	28	1	14
>10	17	17	2	9	55	12	1	9	25	8	1	7

a Protease positions 1 to 99 were analyzed using 109,497 protease sequences, RT positions 1 to 400 were analyzed using 108,848 RT sequences, and integrase positions 1 to 288 were analyzed using 11,778 integrase sequences.

b BLOSUM62 similarity score to the consensus amino acid.

**FIG 2:**
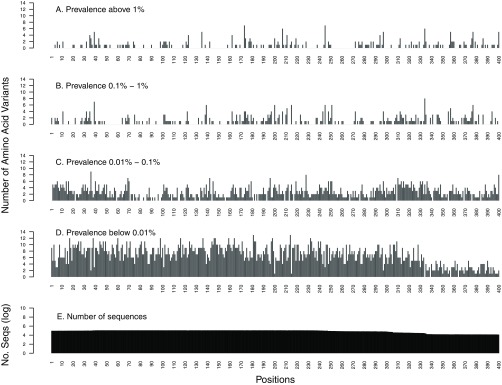
Distribution of the number of HIV-1 reverse transcriptase (RT) amino acid variants by position stratified by prevalence: ≥1% (A), 0.1% to 1% (B), 0.01% to 0.1% (C), and <0.01% (D). The total number of sequences analyzed at each position is shown on a log_10_ scale (E).

Of the 400 RT positions, 147 (37%) had one or more variants occurring at a prevalence of ≥1%, and 240 (60%) had one or more variants in ≥0.1% of sequences (Fig. 2). Overall, there were 636 variants occurring at a prevalence of ≥0.1% at these 240 positions (Table 1).

**FIG 3:**
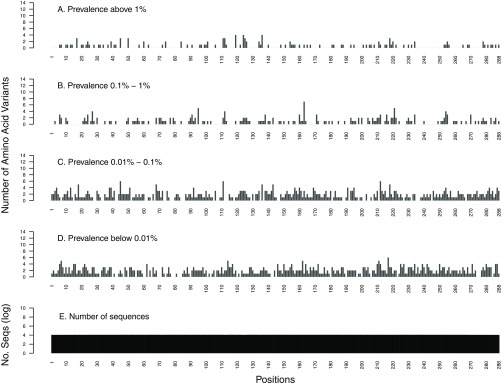
Distribution of the number of HIV-1 integrase (IN) amino acid variants by position stratified by prevalence: ≥1% (A), 0.1% to 1% (B), 0.01% to 0.1% (C), and <0.01% (D). The total number of sequences analyzed at each position is shown on a log_10_ scale (E).

Of the 288 IN positions, 97 (34%) had one or more variants occurring at a prevalence of ≥1%, and 172 (60%) had one or more variants in ≥0.1% of sequences (Fig. 3). Overall, there were 346 variants occurring at a prevalence of ≥0.1% at these 172 positions (Table 1).

### Variability between subtypes.

**FIG 4:**
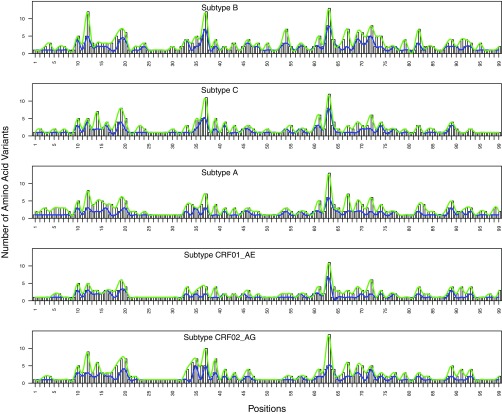
Distribution of the number of HIV-1 protease (PR) amino acid variants present at prevalences of ≥1% (blue) and ≥0.1% (green) by subtype.

**FIG 5:**
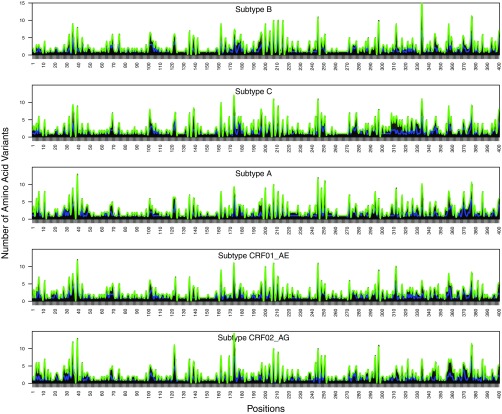
Distribution of the number of HIV-1 reverse transcriptase (RT) amino acid variants present at prevalences of ≥1% (blue) and ≥0.1% (green) by subtype.

**FIG 6:**
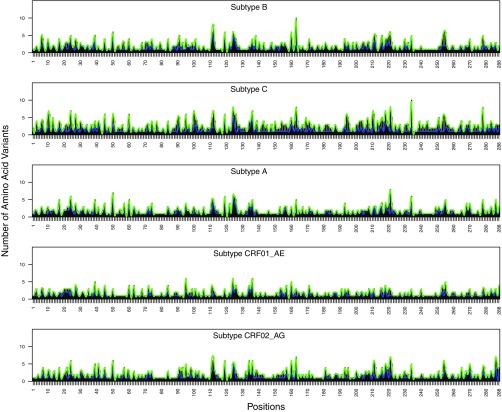
Distribution of the number of HIV-1 integrase (IN) amino acid variants present at prevalences of ≥1% (blue) and ≥0.1% (green) by subtype.

At each position, the number of amino acid variants with a prevalence of ≥0.1% was highly correlated between subtypes: The median intersubtype correlation coefficients for the number of variants with a prevalence above 0.1% were 0.85 (*P* < 2E−16), 0.84 (*P* < 2E−16), and 0.68 (*P* < 2E−16) for PR, RT, and IN, respectively (Fig. 4, 5, and 6).

For amino acid variants with a prevalence of ≥0.1%, the median intersubtype ratio of the prevalence for PR variants was 2.9-fold (interquartile range [IQR], 1.2- to 4.7-fold); only 5.0% of PR variants had a prevalence in one subtype that differed by ≥10-fold in another subtype (range, 10- to 28-fold). The median intersubtype ratio of the prevalence for RT variants was 2.1-fold (IQR, 1.0- to 3.5-fold); only 3.7% of RT variants had a prevalence in one subtype that differed by ≥10-fold in another subtype (range, 10- to 39-fold). The median intersubtype ratio of the prevalence for IN variants was 1.9-fold (IQR, 1.2- to 3.0-fold); only 2.0% of IN variants had a prevalence in one subtype that differed by ≥10-fold in another subtype (range, 10- to 51-fold).

### Chemical relatedness.

There was a strong relationship between the prevalence of an amino acid variant and its biochemical similarity to the consensus amino acid (Table 1). Each 10-fold increase in a variant's prevalence was significantly correlated with the change in BLOSUM62 similarity score: the slopes of a fitted line for each gene were 0.71 (*r* = 0.47; *P* < 2E−16), 0.67 (*r* = 0.41; *P* < 2E−16), and 0.68 (*r* = 0.36; *P* < 2E−16) for PR, RT, and IN, respectively. Similar results were obtained using the BLOSUM80 scoring matrix: the slopes of a fitted line for each gene were 0.81 (*r* = 0.47; *P* < 2E−16), 0.77 (*r* = 0.41; *P* < 2E−16), and 0.74 (*r* = 0.35; *P* < 2E−16) for PR, RT, and IN, respectively.

### Mixture analysis.

There was a strong inverse relationship between a variant's prevalence and the proportion of times that it occurred as part of an electrophoretic mixture. Each 10-fold increase in a variant's prevalence was inversely correlated with the change in the proportion of times that it occurred as part of an electrophoretic mixture: the slopes of a fitted line for each gene were −3.6 (*r* = 0.14; *P* < 2E−06), −5.9 (*r* = 0.32; *P* < 2E−16), and −7.6 (*r* = 0.43; *P* < 2E−16) for PR, RT, and IN, respectively. For example, the very rare variants with a prevalence of <0.01% were present as a part of mixture in 54% to 60% of their occurrences, depending on the gene. In contrast, the most common variants were present as a part of mixture in 7% to 9% of their occurrences, depending on the gene (Table 1).

### Very rare amino acid variants.

The very rare variants occurring at a prevalence of <0.01% were evenly distributed throughout PR, RT, and IN (coefficients of variation [CV], 29% for PR, 43% for RT, and 66% for IN) across positions whether they were highly conserved or were variable at higher-mutation-prevalence strata. In contrast, amino acid variants with higher prevalence had a higher coefficient of variation than variants with lower prevalence: ≥1% (CV, 155% for PR, 179% for RT, and 170% for IN), 0.1% to 1% (CV, 130% for PR, 147% for RT, and 139% for IN), and 0.01% to 0.1% (CV, 73% for PR, 68% for RT, and 76% for IN) (Fig. 1 to 3).

Table S3 in the supplemental material shows that 3.5% of PR, 10.3% of RT, and 6.5% of IN sequences had ≥1 very rare amino acid variant and 0.5% of PR, 2.2% of RT, and 0.9% of IN sequences had ≥2 very rare amino acid variants. The steep reduction in the proportion of sequences with increasing numbers of very rare amino acid variants followed a Poisson distribution.

### Nonpolymorphic TSMs. (i) PR.

To identify nonpolymorphic PI-selected mutations, we analyzed the proportions of all PR mutations in sequences from 61,593 PI-naive individuals and 15,420 PI-experienced individuals. Within PR, 144 mutations at 57 positions were significantly more common in PI-experienced than PI-naive patients after adjustment for multiple-hypothesis testing by controlling the family-wise error rate (i.e., adjusted *P*) at <0.01 (chi-square test; unadjusted *P* < 8.8 × 10^−6^). Of these 144 mutations, 111 at 41 positions were nonpolymorphic and occurred more than five times more frequently in PI-experienced than PI-naive individuals. Table 2 lists each of the 111 nonpolymorphic TSMs by their position and frequency in ARV-experienced individuals.

**TABLE 2:** PI nonpolymorphic treatment-selected mutations

Position	Cons^*a*^	TSM(s)^*b*^	No. of individuals	
			PI treated	PI naïve
10	L	**F**_9.5_ **R**_0.4_ **Y**_0.3_	15,231	60,294
11	V	**L**_0.8_	15,244	60,351
20	K	**T**_5.1_ A_0.1_	15,278	61,114
22	A	**V**_0.9_	15,292	61,145
23	L	**I**_1.2_	15,295	61,252
24	L	**I**_5.9_ **F**_0.6_ **M**_0.2_	15,282	61,263
30	D	**N**_6.3_	15,302	61,316
32	V	**I**_5.1_	15,302	61,323
33	L	**M**_0.1_	15,302	61,317
34	E	**Q**_2.7_ **D**_0.3_ V_0.2_ **N**_0.1_ R_0.1_	15,302	61,315
36	M	A_0.1_	15,296	61,306
38	L	**W**_0.2_	15,304	61,319
43	K	**T**_5.7_ N_0.4_ **I**_0.3_ Q_0.2_ S_0.1_ P_0.04_	15,420	61,587
45	K	Q_0.3_ **I**_0.2_ **V**_0.1_	15,421	61,587
46	M	**I**_22.7_ **L**_10.1_ **V**_0.5_	15,412	61,594
47	I	**V**_4.9_ **A**_0.4_	15,423	61,595
48	G	**V**_4.1_ **M**_0.5_ **A**_0.4_ E_0.2_ **Q**_0.1_ S_0.1_ **L**_0.1_ T_0.05_	15,423	61,597
50	I	**V**_2.0_ **L**_0.5_	15,423	61,597
51	G	**A**_0.3_	15,422	61,592
53	F	**L**_6.0_ **Y**_0.4_ I_0.1_ W_0.1_	15,423	61,598
54	I	**V**_25.5_ **L**_3.2_ **M**_2.8_ **A**_1.4_ **T**_0.9_ **S**_0.7_ C_0.04_	15,422	61,594
55	K	**R**_7.6_ **N**_0.3_	15,421	61,596
66	I	**F**_1.7_ **V**_1.2_ **L**_0.4_	15,423	61,593
67	C	**F**_1.1_ **L**_0.1_	15,418	61,577
71	A	**I**_3.2_ **L**_0.5_	15,415	61,592
72	I	L_2.5_ K_0.7_	15,417	61,574
73	G	**S**_8.7_ **T**_2.6_ **C**_1.2_ **A**_0.7_ **V**_0.2_ D_0.1_ I_0.1_ N_0.05_	15,423	61,592
74	T	**P**_1.9_ **E**_0.1_	15,421	61,591
76	L	**V**_3.8_	15,419	61,585
79	P	**A**_0.9_ N_0.1_	15,421	61,591
82	V	**A**_23.3_ **T**_3.2_ **F**_1.8_ **S**_1.4_ **C**_0.8_ **L**_0.3_ **M**_0.3_ G_0.2_	15,414	61,582
83	N	**D**_0.8_ **S**_0.3_	15,421	61,584
84	I	**V**_14.2_ **A**_0.2_ **C**_0.1_	15,421	61,584
85	I	**V**_4.9_	15,420	61,582
88	N	**D**_5.1_ **S**_1.5_ **G**_0.2_ **T**_0.1_	15,418	61,543
89	L	**V**_4.2_ **T**_0.2_ P_0.1_	15,412	61,533
90	L	**M**_32.0_ I_0.1_	15,416	61,537
91	T	**S**_1.7_ C_0.1_	15,417	61,536
92	Q	**R**_0.9_	15,416	61,527
95	C	**F**_1.7_ **L**_0.2_ **V**_0.1_	15,404	61,251
96	T	S_0.3_	15,391	61,129

a Cons, consensus.

b Nonpolymorphic treatment-selected mutations (TSMs) in boldface were previously reported as being associated with drug resistance (18).

Of the 88 PI nonpolymorphic TSMs that were previously reported by us (18), two mutations, I13M and T74K, were no longer found 5-fold more often in treated compared with untreated individuals. One mutation, Q58E, had a prevalence of 1.1% in subtype D viruses from untreated individuals. The 85 mutations in boldface were previously reported by us as nonpolymorphic TSMs, whereas the remaining 26 mutations are newly identified. Ninety-two percent of the sequences containing a novel nonpolymorphic TSM had one or more PI-associated SDRMs.

### (ii) RT.

To identify nonpolymorphic RTI-selected mutations, we analyzed the proportions of all RT mutations in sequences from 52,040 RTI-naive and 28,806 RTI-experienced individuals. Among the sequences from RTI-naive individuals, 22,810 encompassed RT positions 1 to 300, 4,790 encompassed RT positions 1 to 400, and 2,440 encompassed positions 1 to 560. Among the sequences from RTI-experienced individuals, 14,163 encompassed positions 1 to 300, 5,727 encompassed positions 1 to 400, and 437 encompassed positions 1 to 560.

Within RT, 245 mutations at 116 positions were significantly more common in RTI-experienced than RTI-naive individuals after adjustment for multiple-hypothesis testing by controlling the family-wise error rate (i.e., adjusted *P*) at <0.01 (chi-square test; unadjusted *P* <3.6 × 10^−6^). Of these 245 mutations, 185 mutations at 82 positions were nonpolymorphic and occurred more than five times more frequently in RTI-experienced than RTI-naive individuals. Table 3 lists each of the 95 nonpolymorphic NRTI-selected mutations. Table 4 lists each of the 64 nonpolymorphic NNRTI-selected mutations. Table 5 lists 26 nonpolymorphic RTI-selected mutations that could not be attributed to either NRTI or NNRTI selection pressure alone and that occurred at positions not previously associated with NRTI or NNRTI selection pressure.

**TABLE 3:** NRTI nonpolymorphic treatment-selected mutations

Position	Cons^*a*^	TSM(s)^*b*^	No. of individuals	
			RTI treated	RTI naive
40	E	**F**_0.6_	28,619	51,040
41	M	**L**_28.5_	28,761	51,192
43	K	**N**_1.7_ D_0.1_ H_0.1_	28,768	51,944
44	E	**A**_1.5_	28,769	51,957
64	K	**H**_0.6_ **N**_0.5_ **Y**_0.2_ Q_0.1_	28,796	51,997
65	K	**R**_4.7_ N_0.1_ E_0.1_	28,803	52,000
67	D	**N**_26.8_ **G**_2.5_ **E**_0.5_ **S**_0.3_ **H**_0.2_ **T**_0.2_ A_0.1_ **d**_0.1_	28,792	51,999
68	S	K_0.1_	28,804	52,003
69	T	**D**_6.1_ **i**_0.9_ **G**_0.2_ d_0.2_ **E**_0.2_ Y_0.1_	28,789	52,005
70	K	**R**_18.1_ **E**_0.8_ **G**_0.4_ **T**_0.3_ **N**_0.3_ **Q**_0.3_ **S**_0.1_	28,797	52,013
73	K	M_0.1_	28,804	52,017
74	L	**V**_8.7_ **I**_4.2_	28,799	52,021
75	V	**M**_3.3_ I_3.1_ **T**_1.4_ **A**_0.7_ **S**_0.3_	28,798	52,034
77	F	**L**_1.7_	28,805	52,035
115	Y	**F**_2.3_	28,806	52,037
116	F	**Y**_2.0_	28,807	52,044
117	S	**A**_0.2_	28,802	52,037
151	Q	**M**_2.7_ L_0.2_ K_0.1_	28,792	52,026
157	P	A_0.2_	28,791	52,029
159	I	L_0.1_	28,792	52,027
162	S	D_1.9_	28,763	51,998
164	M	L_0.1_	28,786	52,028
165	T	**L**_0.7_ M_0.1_	28,787	52,021
167	I	**V**_0.6_	28,788	52,020
184	M	**V**_52.5_ **I**_2.5_	28,777	52,016
203	E	K_5.4_ V_0.4_ **A**_0.3_ N_0.1_	28,736	51,864
205	L	F_0.1_	28,738	51,841
208	H	**Y**_7.2_ **F**_0.3_	28,725	51,820
210	L	**W**_17.7_ Y_0.1_ R_0.1_	28,688	51,798
211	R	**D**_0.3_	28,700	51,755
212	W	**M**_0.2_ C_0.1_ L_0.1_	28,705	51,789
215	T	**Y**_26.3_ **F**_10.3_ S_2.1_ **I**_1.9_ N_1.0_ **C**_0.9_ D_0.8_ **V**_0.7_ E_0.2_ G_0.1_ H_0.1_	28,657	51,505
218	D	**E**_5.6_	28,653	51,454
219	K	**Q**_10.9_ **E**_6.1_ **N**_3.1_ **R**_2.7_ **D**_0.3_ **H**_0.3_ **W**_0.3_ G_0.1_ S_0.1_	28,639	51,435
304	A	**G**_0.7_	11,563	19,788

a Cons, consensus.

b Nonpolymorphic treatment-selected mutations (TSMs) in boldface were previously reported as being associated with drug resistance (18). Lowercase “i” indicates an insertion; lowercase “d” indicates a deletion.

**TABLE 4:** NNRTI nonpolymorphic treatment-selected mutations

Position	Cons^*a*^	TSM(s)^*b*^	No. of individuals	
			RTI treated	RTI naive
94	I	**L**_0.6_	28,810	52,041
98	A	**G**_5.7_	28,802	52,042
100	L	**I**_3.6_	28,796	51,999
101	K	**E**_6.6_ **P**_1.3_ **H**_1.1_ **N**_0.4_ T_0.3_ A_0.2_ D_0.1_	28,794	52,039
102	K	**N**_0.4_ G_0.1_	28,804	52,028
103	K	**N**_30.7_ **S**_1.6_ **T**_0.2_ **H**_0.1_	28,805	52,032
105	S	**T**_0.2_	28,808	52,045
106	V	**M**_4.0_ **A**_1.4_	28,805	52,045
108	V	I_7.4_	28,808	52,043
132	I	L_0.7_	28,800	52,037
138	E	**Q**_1.0_ K_0.5_ T_0.1_	28,798	52,024
139	T	**R**_0.8_	28,798	52,037
178	I	**F**_0.2_	28,781	52,001
179	V	**F**_0.2_ L_0.1_ M_0.1_	28,774	52,010
181	Y	**C**_16.6_ **I**_0.7_ **V**_0.5_ F_0.2_ G_0.1_ N_0.1_	28,780	52,016
188	Y	**L**_3.7_ **C**_0.8_ **H**_0.7_ F_0.4_	28,758	52,014
190	G	**A**_12.7_ **S**_2.3_ **E**_0.4_ **Q**_0.3_ **C**_0.1_	28,771	52,015
221	H	**Y**_6.1_ **C**_0.1_	28,565	50,963
225	P	**H**_3.7_	28,386	50,583
227	F	**L**_2.3_ **Y**_0.2_	28,165	50,128
230	M	**L**_1.4_	28,081	49,720
232	Y	**H**_0.3_	27,827	49,437
234	L	**I**_0.2_	27,760	49,216
238	K	**T**_1.9_ **N**_0.4_	27,404	47,232
240	T	K_0.1_	23,831	46,204
241	V	**M**_0.2_	23,586	44,549
242	Q	H_0.9_ L_0.2_ **K**_0.1_	23,529	43,984
318	Y	**F**_1.3_	10,809	15,668
348	N	**I**_13.0_ T_0.8_	6,367	5,528
404	E	N_1.3_	1,207	3,663

a Cons, consensus.

b Nonpolymorphic treatment-selected mutations (TSMs) in boldface were previously reported as being associated with drug resistance (18).

**TABLE 5:** Undifferentiated RTI nonpolymorphic treatment-selected mutations

Position	Cons^*a*^	TSM(s)^*b*^	No. of individuals	
			RTI treated	RTI naive
3	S	**C**_0.3_	19,241	42,633
16	M	V_0.4_	19,884	43,640
31	I	**L**_1.6_	21,490	45,863
33	A	V_0.2_	21,573	46,050
34	L	I_0.7_	21,582	46,129
54	N	I_0.1_	28,794	51,991
58	T	**N**_0.2_ S_0.2_	28,795	51,994
109	L	**I**_0.8_ M_0.1_ **V**_0.1_	28,808	52,043
202	I	T_0.1_	28,742	51,873
223	K	Q_2.1_ E_1.7_ **T**_0.5_ P_0.1_	28,537	50,880
228	L	R_5.4_ **N**_0.1_ I_0.1_ K_0.1_	28,148	50,071
302	E	D_0.3_	12,507	20,464
312	E	G_0.4_	10,935	17,751
341	I	F_1.4_	6,671	5,802
394	Q	S_0.8_	6,108	4,874
399	E	G_1.2_	5,882	4,830
547	Q	R_3.6_	473	2,559

a Cons, consensus.

b Nonpolymorphic treatment-selected mutations (TSMs) in boldface were previously reported as being associated with drug resistance (18).

Of the 122 RTI nonpolymorphic TSMs that were previously reported by us (18), two mutations, P236L and D237E, were no longer found to be 5-fold more common in treated compared with untreated individuals. One mutation, K43Q, was found to have a prevalence of 2.0% in CRF01_AE viruses from ARV-naive individuals, and another mutation, L228H, was found to have a prevalence of 1.2% in subtype F viruses from ARV-naive individuals. In Tables 3, 4, and 5, the 118 mutations shown in boldface were previously reported by us to be nonpolymorphic TSMs, whereas the remaining 63 are newly identified. Ninety-eight percent of the sequences containing a novel nonpolymorphic TSM in RTI-experienced individuals had one or more RTI-associated SDRMs.

### (iii) IN.

To identify nonpolymorphic INSTI-selected mutations, we analyzed the proportions of all IN mutations in sequences from 6,630 INSTI-naive and 1,020 INSTI-experienced individuals. Within IN, 45 mutations at 28 positions were significantly more common in INSTI-experienced than INSTI-naive individuals after adjustment for multiple-hypothesis testing by controlling the family-wise error rate (i.e., adjusted *P*) at <0.01 (chi-square test; unadjusted *P* <1.3 × 10^−5^). Of these 45 mutations, 44 occurred more than five times more frequently in INSTI-experienced than INSTI-naive individuals. Of these 44 TSMs, 30 at 15 positions were nonpolymorphic in INSTI-naive patients. Table 6 shows those 30 nonpolymorphic TSMs. Of these 30 nonpolymorphic TSMs, 23 in boldface are established previously reported DRMs (23), and the remaining 7 were new: V79I, E92A, E138T, P142T, Q148N, N155D, and D253Y. Eighty-one percent of the sequences containing a novel nonpolymorphic TSM had one or more established INSTI-associated DRMs.

**TABLE 6:** INSTI nonpolymorphic treatment-selected mutations

Position	Cons^*a*^	TSM(s)^*b*^	No. of individuals	
			INSTI treated	INSTI naive
51	H	**Y**_0.5_	1,019	6,609
66	T	**I**_1.3_ **A**_0.7_ **K**_0.4_	1,019	6,619
79	V	I_2.5_	1,020	6,625
92	E	**Q**_6.4_ A_0.4_	1,020	6,628
95	Q	**K**_1.6_	1,020	6,627
121	F	**Y**_0.4_	1,020	6,631
138	E	**K**_5.9_ **A**_3.0_ T_0.7_	1,020	6,631
140	G	**S**_25.2_ **A**_2.1_ **C**_0.7_	1,020	6,631
142	P	T_0.6_	1,020	6,631
143	Y	**R**_7.7_ **C**_5.4_ **H**_2.8_ **S**_0.6_ **G**_0.4_	1,020	6,631
147	S	**G**_1.6_	1,020	6,631
148	Q	**H**_22.6_ **R**_7.9_ **K**_1.0_ N_0.4_	1,020	6,629
155	N	**H**_30.8_ D_0.5_	1,020	6,629
230	S	**R**_3.6_	1,018	6,608
253	D	Y_1.0_	1,018	6,588

a Cons, consensus.

b Nonpolymorphic treatment-selected mutations (TSMs) in boldface were previously reported as being associated with drug resistance (9).

### Synonymous and nonsynonymous mutation rates.

Among the 99 PR positions, *dN* was higher than *dS* at a median of 18 positions in the five most common subtypes. *dN* was higher than *dS* in all five subtypes at positions 12, 13, 15, and 37. Among the 400 RT positions studied for amino acid variation, *dN* was higher than *dS* at a median of 37 positions in the five most common subtypes. *dN* was higher than *dS* in all five subtypes at positions 35, 135, 178, 200, 202, 272, and 369. Among the 288 IN positions, *dN* was higher than *dS* at a median of 28 positions in the five most common subtypes. *dN* was higher than *dS* in all five subtypes at positions 124 and 218.

Among the PR TSMs, the minimum numbers of nucleotide differences between the TSM and the consensus amino acid variant were 1 for 67.6% and 2 for 32.4% (i.e., these were 2-bp mutations). Among the RT TSMs, the minimum numbers of nucleotide differences were 1 for 68.4%, 2 for 31.1%, and 3 for 0.6%. Among the IN TSMs, the minimum numbers of nucleotide differences were 1 for 86.7% and 2 for 13.3%.

## DISCUSSION

Within an individual, HIV-1 variation arises from repeated cycles of virus polymerization errors, recombination, APOBEC-mediated RNA editing, and selective drug and immune pressure (24, 25). Although HIV-1 has a high mutation rate, only those variants without significantly impaired fitness will rise to levels detectable by standard direct PCR Sanger sequencing. In contrast, it is expected that many virus polymerization errors will result in nonviable variants or variants that may not compete successfully with more-fit virus variants (26). The consistent presence of certain mutations by Sanger sequencing attests to their fitness at least under some conditions and genetic contexts.

An extensive amount of data are available for characterizing HIV-1 PR, RT, and IN variability because these genes are frequently sequenced for clinical, research, and epidemiological purposes. We analyzed PR and RT sequences from more than 100,000 individuals and IN sequences from more than 10,000 individuals and identified 1,183 amino acid variants in PR, RT, and IN that were present in ≥0.1% of sequences. We also analyzed several subsets of these sequences from individuals with known ARV treatment histories and identified 326 nonpolymorphic PR, RT, and IN TSMs.

### Overall PR, RT, and IN variability.

Forty-seven percent of PR, 37% of RT, and 34% of IN positions had one or more amino acid variants with a prevalence of ≥1%. Seventy percent of PR, 60% of RT, and 60% of IN positions had one or more amino acid variants with a prevalence of ≥0.1%. Although amino acid variants occurred in different proportions in different subtypes, the prevalence of a variant in one subtype rarely differed by more than 10-fold compared with the prevalence of that variant in a different subtype (2.0% of IN variants, 3.7% of RT variants, and 5.0% of PR variants).

In each gene, the more rare the amino acid variant, the more likely it was present as part of an electrophoretic mixture or differed biochemically from the consensus amino acid. Variants that occur frequently as part of electrophoretic mixtures are likely to have reduced replication fitness, explaining their inability to replicate sufficiently to become dominant within an infected individual's circulating virus population (27, 28). Although the presence of two electrophoretic peaks at a position is usually a reliable indicator that two nucleotides are present in that virus population, a small secondary peak can also result from PCR error and sequencing artifact (29, 30).

Very rare variants had the lowest biochemical similarity to the consensus amino acid at each position and often occurred as part of an electrophoretic mixture. Additionally, these variants were evenly distributed across all positions in PR, RT, and IN—occurring in similar numbers at positions that were highly conserved or displayed variability at higher mutation thresholds. We propose that it is useful to identify sequences that contain large numbers of such rare variants because a high number of very rare amino acids in a direct PCR dideoxynucleotide terminator Sanger sequence could result from sequencing error or unrecognized frameshifts if the rare amino acids are clustered. Additionally, the presence of a high number of very rare variants in a next-generation deep-sequencing assay would be more consistent with PCR error than quasispecies variation and would suggest that the threshold for identification of low-abundance variants was set too low.

### Treatment-selected mutations.

We previously published an analysis of nonpolymorphic TSMs in PR and the first 350 positions of RT using an earlier data set containing sequences from approximately 25,000 individuals with known ARV treatment histories (18). In this article, we extended our analysis of nonpolymorphic TSMs to IN and to the entire RT. In addition, the numbers of sequences from individuals with known treatment histories in PR and the 5′ part of RT were nearly three times higher for PR and RT than those in our previous analysis.

We identified 111 nonpolymorphic PR TSMs: 26 new TSMs and 85 of the 88 previously identified TSMs. The novel PR TSMs are likely to be accessory drug resistance mutations because they nearly always occurred in combination with established PI resistance mutations.

We identified 185 nonpolymorphic RT TSMs: 67 new TSMs and 118 of the 122 previously identified TSMs. The novel RT TSMs were likely to be accessory drug resistance mutations because they nearly always occurred in combination with established NRTI or NNRTI resistance mutations.

Of the 185 RT TSMs, 95 were selected by NRTIs and 64 were selected by NNRTIs. For 26 RT TSMs, however, it was not possible to determine whether the mutations were primarily selected by NRTIs or NNRTIs because most of the individuals with these 26 TSMs received both NRTIs and NNRTIs.

Several mutations in the connection and RNase H domains of RT have been shown to play an accessory role in reducing HIV-1 susceptibility in combination with thymidine analog mutations (TAMs), most likely by slowing the activity of RNase H and thereby allowing more time for TAM-mediated primer unblocking (31). However, only 11 TSMs were identified beyond position 300, including the NRTI-selected mutation A304G, the NNRTI-selected mutations Y318F, N348IT, and E404N, and the RTI-selected mutations E302D, E312G, I341F, Q394S, E399G, and Q547G. This is consistent with the much lower number of sequenced viruses extending beyond position 300 obtained from NRTI- and/or NNRTI-experienced individuals.

We identified 30 nonpolymorphic IN TSMs, including 23 established INSTI resistance mutations (H51Y, T66IAK, E92Q, Q95K, F121Y, E138KA, G140SAC, Y143RCHSG, S147G, Q148HRK, N155H, and S230R) and seven novel mutations not previously associated with INSTI resistance. Four of the novel mutations—E92A, E138T, Q148N, and N155D—were at positions also containing established INSTI resistance mutations. Three other mutations—V79I, P142T, and D253Y—were at novel positions. Eighty-two percent of the sequences containing one of these three novel nonpolymorphic TSMs had one or more established INSTI-associated DRMs.

Four well-characterized accessory INSTI-associated DRMs—L74M, T97A, and G163R/K—were not identified because they were polymorphic in one or more subtypes (32). G118R and R263K, two other highly studied mutations (21, 33), were also not identified. G118R is extremely rare and was not present in a single plasma virus sequence. R263K was significantly more common in INSTI-treated than INSTI-naive sequences (6/1,016 [0.59%] versus 8/6558 [0.12%]), but this difference was not significant after controlling for multiple comparisons.

Although practically all major drug resistance mutations are TSMs, the converse may not always be true. For example, many TSMs are accessory mutations that only arise in the presence of other drug resistance mutations. Other TSMs such as the T215 revertant mutations T215S/C/E/D/I/V have been shown to arise from drug resistance mutations (e.g., T215Y/F) when selective drug pressure is removed (34).

### APOBEC.

We previously published an analysis of mutations indicative of APOBEC-mediated RNA editing that encompassed PR and the first 240 positions of RT (13). Our current analysis identified two new mutations in PR and one new mutation in the previously analyzed region of RT. Additionally, we identified 55 mutations between RT positions 241 and 560 and 71 mutations in IN that are also likely to result from APOBEC-mediated RNA editing. We then predicted that most sequences with two or more of these mutations were likely to have undergone G-to-A hypermutation.

Identification of sequences with G-to-A hypermutation is important because the extent of hypermutation is usually incomplete and may not be uniformly distributed (13, 35, 36) and because several mutations known to emerge from selective drug pressure can also arise from G-to-A hypermutation, including D30N, M46I, and G73S in PR, D67N, E138K, M184I, G190SE, and M230I in RT, and E138K, G118R, and G163R in IN. As drug resistance testing in low- and middle-income countries will increasingly be performed using dried blood spots, which often contain proviral HIV-1 DNA (36–39), it will become necessary to determine if a sequence has evidence of G-to-A hypermutation to assess the clinical significance of the above drug resistance mutations. For example, the isolated presence of DRMs associated with G-to-A hypermutation would need to be judged differently if they occurred in a sequence containing an excess of the APOBEC-indicating mutations that we describe in this study.

### Conclusions.

This study of HIV-1 PR, RT, and IN variability makes it possible to apportion amino acid variants into the following categories: (i) established variants that may or may not be a nonpolymorphic TSM, (ii) APOBEC-associated mutations, and (iii) very rare variants of questionable validity or replication potential.

Determination of whether a particular sequence contains an excess of APOBEC-associated mutations or of very rare amino acid variants can be used to optimally determine the significance of other mutations present in that sequence, particularly when that sequence is generated using technologies associated with greater sequencing artifacts, as occurs with the use of samples likely to be enriched for proviral DNA or with NGS deep sequencing. As the number of sequences for IN and the 3′ part of RT was approximately 10-fold lower than those for PR and the 5′ part of RT and as subtype B was overly represented in our data set, we will update our estimates of the prevalence of each mutation at each position as additional sequence data are available.

## Supplementary Material

Supplemental material

## References

[1] Li G, Piampongsant S, Faria NR, Voet A, Pineda-Pena AC, Khouri R, Lemey P, Vandamme AM, Theys K 2015 An integrated map of HIV genome-wide variation from a population perspective. Retrovirology 12:18. doi:10.1186/s12977-015-0148-6. 25808207 PMC4358901

[2] Abram ME, Ferris AL, Shao W, Alvord WG, Hughes SH 2010 Nature, position, and frequency of mutations made in a single cycle of HIV-1 replication. J Virol 84:9864–9878. doi:10.1128/JVI.00915-10. 20660205 PMC2937799

[3] Onafuwa-Nuga A, Telesnitsky A 2009 The remarkable frequency of human immunodeficiency virus type 1 genetic recombination. Microbiol Mol Biol Rev 73:451–480. doi:10.1128/MMBR.00012-09. 19721086 PMC2738136

[4] Loeb DD, Swanstrom R, Everitt L, Manchester M, Stamper SE, Hutchison CA III 1989 Complete mutagenesis of the HIV-1 protease. Nature 340:397–400. doi:10.1038/340397a0. 2666861

[5] Rihn SJ, Hughes J, Wilson SJ, Bieniasz PD 2015 Uneven genetic robustness of HIV-1 integrase. J Virol 89:552–567. doi:10.1128/JVI.02451-14. 25339768 PMC4301135

[6] Smith RA, Loeb LA, Preston BD 2005 Lethal mutagenesis of HIV. Virus Res 107:215–228. doi:10.1016/j.virusres.2004.11.011. 15649567

[7] Keys JR, Zhou S, Anderson JA, Eron JJ Jr, Rackoff LA, Jabara C, Swanstrom R 2015 Primer ID informs next-generation sequencing platforms and reveals preexisting drug resistance mutations in the HIV-1 reverse transcriptase coding domain. AIDS Res Hum Retroviruses 31:658–668. doi:10.1089/aid.2014.0031. 25748056 PMC4458739

[8] Shao W, Boltz VF, Spindler JE, Kearney MF, Maldarelli F, Mellors JW, Stewart C, Volfovsky N, Levitsky A, Stephens RM, Coffin JM 2013 Analysis of 454 sequencing error rate, error sources, and artifact recombination for detection of low-frequency drug resistance mutations in HIV-1 DNA. Retrovirology 10:18. doi:10.1186/1742-4690-10-18. 23402264 PMC3599717

[9] Rhee SY, Gonzales MJ, Kantor R, Betts BJ, Ravela J, Shafer RW 2003 Human immunodeficiency virus reverse transcriptase and protease sequence database. Nucleic Acids Res 31:298–303. doi:10.1093/nar/gkg100. 12520007 PMC165547

[10] Pineda-Pena AC, Faria NR, Imbrechts S, Libin P, Abecasis AB, Deforche K, Gomez-Lopez A, Camacho RJ, de Oliveira T, Vandamme AM 2013 Automated subtyping of HIV-1 genetic sequences for clinical and surveillance purposes: performance evaluation of the new REGA version 3 and seven other tools. Infect Genet Evol 19:337–348. doi:10.1016/j.meegid.2013.04.032. 23660484

[11] Learn GH Jr, Korber BT, Foley B, Hahn BH, Wolinsky SM, Mullins JI 1996 Maintaining the integrity of human immunodeficiency virus sequence databases. J Virol 70:5720–5730. 8764096 10.1128/jvi.70.8.5720-5730.1996 PMC190542

[12] Fourati S, Malet I, Lambert S, Soulie C, Wirden M, Flandre P, Fofana DB, Sayon S, Simon A, Katlama C, Calvez V, Marcelin AG 2012 E138K and M184I mutations in HIV-1 reverse transcriptase coemerge as a result of APOBEC3 editing in the absence of drug exposure. AIDS 26:1619–1624. doi:10.1097/QAD.0b013e3283560703. 22695298

[13] Gifford RJ, Rhee SY, Eriksson N, Liu TF, Kiuchi M, Das AK, Shafer RW 2008 Sequence editing by apolipoprotein B RNA-editing catalytic component-B and epidemiological surveillance of transmitted HIV-1 drug resistance. AIDS 22:717–725. doi:10.1097/QAD.0b013e3282f5e07a. 18356601 PMC2946849

[14] Mangeat B, Turelli P, Caron G, Friedli M, Perrin L, Trono D 2003 Broad antiretroviral defence by human APOBEC3G through lethal editing of nascent reverse transcripts. Nature 424:99–103. doi:10.1038/nature01709. 12808466

[15] Cornish-Bowden A 1985 Nomenclature for incompletely specified bases in nucleic acid sequences: recommendations 1984. Nucleic Acids Res 13:3021–3030. doi:10.1093/nar/13.9.3021. 2582368 PMC341218

[16] Holm S 1979 A simple sequentially rejective multiple test procedure. Scand J Stat 6:65–70.

[17] Bennett DE, Camacho RJ, Otelea D, Kuritzkes DR, Fleury H, Kiuchi M, Heneine W, Kantor R, Jordan MR, Schapiro JM, Vandamme AM, Sandstrom P, Boucher CA, van de Vijver D, Rhee SY, Liu TF, Pillay D, Shafer RW 2009 Drug resistance mutations for surveillance of transmitted HIV-1 drug-resistance: 2009 update. PLoS One 4:e4724. doi:10.1371/journal.pone.0004724. 19266092 PMC2648874

[18] Shahriar R, Rhee SY, Liu TF, Fessel WJ, Scarsella A, Towner W, Holmes SP, Zolopa AR, Shafer RW 2009 Nonpolymorphic human immunodeficiency virus type 1 protease and reverse transcriptase treatment-selected mutations. Antimicrob Agents Chemother 53:4869–4878. doi:10.1128/AAC.00592-09. 19721070 PMC2772298

[19] Ashkenazy H, Penn O, Doron-Faigenboim A, Cohen O, Cannarozzi G, Zomer O, Pupko T 2012 FastML: a web server for probabilistic reconstruction of ancestral sequences. Nucleic Acids Res 40:W580–W584. doi:10.1093/nar/gks498. 22661579 PMC3394241

[20] Azijn H, Tirry I, Vingerhoets J, de Bethune MP, Kraus G, Boven K, Jochmans D, Van Craenenbroeck E, Picchio G, Rimsky LT 2010 TMC278, a next-generation nonnucleoside reverse transcriptase inhibitor (NNRTI), active against wild-type and NNRTI-resistant HIV-1. Antimicrob Agents Chemother 54:718–727. doi:10.1128/AAC.00986-09. 19933797 PMC2812151

[21] Quashie PK, Mesplede T, Han YS, Veres T, Osman N, Hassounah S, Sloan RD, Xu HT, Wainberg MA 2013 Biochemical analysis of the role of G118R-linked dolutegravir drug resistance substitutions in HIV-1 integrase. Antimicrob Agents Chemother 57:6223–6235. doi:10.1128/AAC.01835-13. 24080645 PMC3837891

[22] Malet I, Fourati S, Charpentier C, Morand-Joubert L, Armenia D, Wirden M, Sayon S, Van Houtte M, Ceccherini-Silberstein F, Brun-Vezinet F, Perno CF, Descamps D, Capt A, Calvez V, Marcelin AG 2011 The HIV-1 integrase G118R mutation confers raltegravir resistance to the CRF02_AG HIV-1 subtype. J Antimicrob Chemother 66:2827–2830. doi:10.1093/jac/dkr389. 21933786

[23] Wensing AM, Calvez V, Gunthard HF, Johnson VA, Paredes R, Pillay D, Shafer RW, Richman DD 2014 2014 update of the drug resistance mutations in HIV-1. Top Antivir Med 22:642–650. 25101529 PMC4392881

[24] Rambaut A, Posada D, Crandall KA, Holmes EC 2004 The causes and consequences of HIV evolution. Nat Rev Genet 5:52–61. doi:10.1038/nrg1246. 14708016

[25] Wood N, Bhattacharya T, Keele BF, Giorgi E, Liu M, Gaschen B, Daniels M, Ferrari G, Haynes BF, McMichael A, Shaw GM, Hahn BH, Korber B, Seoighe C 2009 HIV evolution in early infection: selection pressures, patterns of insertion and deletion, and the impact of APOBEC. PLoS Pathog 5:e1000414. doi:10.1371/journal.ppat.1000414. 19424423 PMC2671846

[26] Coffin JM 1995 HIV population dynamics in vivo: implications for genetic variation, pathogenesis, and therapy. Science 267:483–489. doi:10.1126/science.7824947. 7824947

[27] Fourati S, Visseaux B, Armenia D, Morand-Joubert L, Artese A, Charpentier C, Van Den Eede P, Costa G, Alcaro S, Wirden M, Perno CF, Ceccherini Silberstein F, Descamps D, Calvez V, Marcelin AG 2013 Identification of a rare mutation at reverse transcriptase Lys65 (K65E) in HIV-1-infected patients failing on nucleos(t)ide reverse transcriptase inhibitors. J Antimicrob Chemother 68:2199–2204. doi:10.1093/jac/dkt200. 23749955

[28] Garcia-Lerma JG, Gerrish PJ, Wright AC, Qari SH, Heneine W 2000 Evidence of a role for the Q151L mutation and the viral background in development of multiple dideoxynucleoside-resistant human immunodeficiency virus type 1. J Virol 74:9339–9346. doi:10.1128/JVI.74.20.9339-9346.2000. 11000201 PMC112361

[29] Huang DD, Eshleman SH, Brambilla DJ, Palumbo PE, Bremer JW 2003 Evaluation of the editing process in human immunodeficiency virus type 1 genotyping. J Clin Microbiol 41:3265–3272. doi:10.1128/JCM.41.7.3265-3272.2003. 12843074 PMC165383

[30] Woods CK, Brumme CJ, Liu TF, Chui CK, Chu AL, Wynhoven B, Hall TA, Trevino C, Shafer RW, Harrigan PR 2012 Automating HIV drug resistance genotyping with RECall, a freely accessible sequence analysis tool. J Clin Microbiol 50:1936–1942. doi:10.1128/JCM.06689-11. 22403431 PMC3372133

[31] Delviks-Frankenberry KA, Nikolenko GN, Pathak VK 2010 The “connection” between HIV drug resistance and RNase H. Viruses 2:1476–1503. doi:10.3390/v2071476. 21088701 PMC2982141

[32] Llacer Delicado T, Torrecilla E, Holguin A 2016 Deep analysis of HIV-1 natural variability across HIV-1 variants at residues associated with integrase inhibitor (INI) resistance in INI-naive individuals. J Antimicrob Chemother 71:362–366. doi:10.1093/jac/dkv333. 26546669

[33] Quashie PK, Mesplede T, Han YS, Oliveira M, Singhroy DN, Fujiwara T, Underwood MR, Wainberg MA 2012 Characterization of the R263K mutation in HIV-1 integrase that confers low-level resistance to the second-generation integrase strand transfer inhibitor dolutegravir. J Virol 86:2696–2705. doi:10.1128/JVI.06591-11. 22205735 PMC3302270

[34] Yerly S, Rakik A, De Loes SK, Hirschel B, Descamps D, Brun-Vezinet F, Perrin L 1998 Switch to unusual amino acids at codon 215 of the human immunodeficiency virus type 1 reverse transcriptase gene in seroconvertors infected with zidovudine-resistant variants. J Virol 72:3520–3523. 9557630 10.1128/jvi.72.5.3520-3523.1998 PMC109570

[35] Pace C, Keller J, Nolan D, James I, Gaudieri S, Moore C, Mallal S 2006 Population level analysis of human immunodeficiency virus type 1 hypermutation and its relationship with APOBEC3G and *vif* genetic variation. J Virol 80:9259–9269. doi:10.1128/JVI.00888-06. 16940537 PMC1563905

[36] Kieffer TL, Kwon P, Nettles RE, Han Y, Ray SC, Siliciano RF 2005 G→A hypermutation in protease and reverse transcriptase regions of human immunodeficiency virus type 1 residing in resting CD4^+^ T cells in vivo. J Virol 79:1975–1980. doi:10.1128/JVI.79.3.1975-1980.2005. 15650227 PMC544145

[37] Sanchez G, Xu X, Chermann JC, Hirsch I 1997 Accumulation of defective viral genomes in peripheral blood mononuclear cells of human immunodeficiency virus type 1-infected individuals. J Virol 71:2233–2240. 9032358 10.1128/jvi.71.3.2233-2240.1997 PMC191331

[38] Hamers RL, Smit PW, Stevens W, Schuurman R, Rinke de Wit TF 2009 Dried fluid spots for HIV type-1 viral load and resistance genotyping: a systematic review. Antivir Ther 14:619–629. 19704164

[39] Parkin NT 2014 Measurement of HIV-1 viral load for drug resistance surveillance using dried blood spots: literature review and modeling of contribution of DNA and RNA. AIDS Rev 16:160–171. 25221990

