# Supplementary material for: HIV-1 Protease, Reverse Transcriptase, and Integrase Variation
Source: J Virol. 2016 Jun 10;90(13):6058–70. doi: 10.1128/JVI.00495-16 (PMC4907232; doi:10.1128/JVI.00495-16)
Supplement: Supplemental material [file supp_90_13_6058__index.html]

Supplemental material 

# HIV-1 Protease, Reverse Transcriptase, and Integrase Variation

## Supplemental material

- Supplemental file 1 -

  Table S1 (List of mutations indicative of APOBEC-mediated G-to-A editing.)

  Table S2 (Estimated proportion of APOBEC-edited sequences according to number of signature APOBEC-associated mutations.)

  Table S3 (Numbers of sequences with very rare amino acid variants occurring at a prevalence <0.01%.)

  PDF, 124K
