# Supplementary material for: HIV-1 Protease, Reverse Transcriptase, and Integrase Variation
Source: J Virol. 2016 Jun 10;90(13):6058–70. doi: 10.1128/JVI.00495-16 (PMC4907232; doi:10.1128/JVI.00495-16)
Supplement: Supplemental material [file JVI.00495-16_zjv999181745so1.pdf]

Supplementary Table 1. List of Mutations Indicative of APOBEC-Mediated G-to-A Editing

Gene	Position	WT	NA	Target Codon Position	APOBEC	Signature APOBEC Mutation	No. Plasma Samples
PR	6	W	TGG	2	3G	*	5
PR	17	G	GG-	1	3F, 3G	K	0
PR	21	E	GA-	1	3F	N	6
PR	25	D	GA-	1	3F	K	4
PR	25	D	GA-	1	3F	N	29
PR	27	G	GG-	1	3F, 3G	E	8
PR	27	G	GG-	1	3F, 3G	K	0
PR	27	G	GG-	1	3G	R	7
PR	27	G	GG-	1	3G	S	1
PR	29	D	GAT	1	3F	K	0
PR	29	D	GAT	1	3F	N	22
PR	30	D	GA-	1	3F	K	0
PR	40	G	GG-	1	3F, 3G	E	22
PR	40	G	GG-	1	3G	R	17
PR	40	G	GG-	1	3G	S	0
PR	42	W	TGG	2, 3	3F, 3G	*	13
PR	48	G	GG-	1	3F, 3G	E	29
PR	48	G	GG-	1	3F, 3G	K	2
PR	48	G	GG-	1	3G	R	19
PR	49	G	GGA	1, 2	3F	E	27
PR	49	G	GGA	1, 2	3F	K	2
PR	49	G	GGA	1	3G	R	11
PR	51	G	GG-	1	3F, 3G	E	16
PR	51	G	GG-	1	3F, 3G	K	8
PR	51	G	GG-	1	3G	R	14
PR	51	G	GG-	1	3G	S	0
PR	52	G	GG-	1	3F, 3G	E	4
PR	52	G	GG-	1	3F, 3G	K	1
PR	52	G	GG-	1	3G	R	6
PR	52	G	GG-	1	3G	S	8
PR	68	G	GG-	1	3F, 3G	K	2
PR	68	G	GG-	1	3G	R	13
PR	68	G	GG-	1	3G	S	0
PR	78	G	GG-	1	3F, 3G	E	8
PR	78	G	GG-	1	3F, 3G	K	1
PR	78	G	GG-	1	3G	R	24
PR	86	G	GG-	1	3F, 3G	E	17
PR	86	G	GG-	1	3F, 3G	K	4
PR	86	G	GG-	1	3G	R	26
PR	86	G	GG-	1	3G	S	0
PR	94	G	GG-	1	3F, 3G	K	0
PR	94	G	GG-	1	3G	R	6
PR	94	G	GG-	1	3G	S	13
RT	15	G	GG-	1	3F, 3G	K	7
RT	15	G	GG-	1	3G	R	30

RT	15	G	GG-	1	3G	S	0
RT	16	M	ATG	3	3G	I	25
RT	17	D	GAT	1	3F	N	24
RT	18	G	GG-	1	3F, 3G	K	0
RT	18	G	GG-	1	3G	R	8
RT	18	G	GG-	1	3G	S	2
RT	24	W	TGG	2	3G	*	9
RT	41	M	-TG	3	3G	I	34
RT	42	E	GA-	1	3F	K	67
RT	42	E	GA-	1	3F	N	0
RT	45	G	GG-	1	3F, 3G	K	5
RT	45	G	GG-	1	3G	R	95
RT	51	G	GG-	1	3F, 3G	K	2
RT	51	G	GG-	1	3G	R	27
RT	53	E	GA-	1	3F	K	41
RT	71	W	TGG	2, 3	3F, 3G	*	11
RT	76	D	GA-	1	3F	K	0
RT	79	E	GA-	1	3F	K	20
RT	86	D	GA-	1	3F	K	0
RT	88	W	TGG	2, 3	3F, 3G	*	17
RT	89	E	GA-	1	3F	K	17
RT	89	E	GA-	1	3F	N	0
RT	93	G	GG-	1	3F, 3G	E	10
RT	93	G	GG-	1	3F, 3G	K	4
RT	93	G	GG-	1	3G	R	23
RT	99	G	GG-	1	3F, 3G	E	6
RT	99	G	GG-	1	3F, 3G	K	0
RT	99	G	GG-	1	3G	R	29
RT	99	G	GG-	1	3G	S	2
RT	110	D	GAT	1	3F	K	0
RT	110	D	GAT	1	3F	N	16
RT	112	G	GG-	1	3F, 3G	E	13
RT	112	G	GG-	1	3F, 3G	K	3
RT	112	G	GG-	1	3G	R	0
RT	112	G	GG-	1	3G	S	13
RT	113	D	GA-	1	3F	K	2
RT	141	G	GG-	1	3F, 3G	E	20
RT	141	G	GG-	1	3F, 3G	K	4
RT	141	G	GG-	1	3G	R	32
RT	141	G	GG-	1	3G	S	0
RT	152	G	GG-	1	3F, 3G	E	18
RT	152	G	GG-	1	3F, 3G	K	0
RT	152	G	GG-	1	3G	R	12
RT	152	G	GG-	1	3G	S	0
RT	153	W	TGG	2, 3	3F, 3G	*	19
RT	155	G	GG-	1	3F, 3G	E	10
RT	155	G	GG-	1	3F, 3G	K	1
RT	155	G	GG-	1	3G	R	20
RT	155	G	GG-	1	3G	S	0

RT	185	D	GAT	1	3F	K	0
RT	185	D	GAT	1	3F	N	11
RT	186	D	GA-	1	3F	K	0
RT	186	D	GA-	1	3F	N	22
RT	192	D	GA-	1	3F	K	0
RT	212	W	TGG	2, 3	3F, 3G	*	16
RT	213	G	GG-	1	3F, 3G	K	3
RT	213	G	GG-	1	3G	R	15
RT	229	W	TGG	2, 3	3F, 3G	*	15
RT	231	G	GG-	1	3F, 3G	K	2
RT	231	G	GG-	1	3G	R	8
RT	231	G	GG-	1	3G	S	5
RT	233	E	GA-	1	3F	K	21
RT	239	W	TGG	2, 3	3F, 3G	*	3
RT	252	W	TGG	2, 3	3F, 3G	*	9
RT	256	D	GA-	1	3F	K	0
RT	256	D	GA-	1	3F	N	5
RT	262	G	GG-	1	3F, 3G	K	2
RT	262	G	GG-	1	3G	R	12
RT	262	G	GG-	1	3G	S	0
RT	266	W	TGG	2, 3	3F, 3G	*	9
RT	273	G	GG-	1	3F, 3G	E	7
RT	273	G	GG-	1	3F, 3G	K	7
RT	273	G	GG-	1	3G	S	0
RT	285	G	GG-	1	3F, 3G	E	9
RT	285	G	GG-	1	3F, 3G	K	1
RT	285	G	GG-	1	3G	R	8
RT	298	E	GA-	1	3F	N	0
RT	302	E	GA-	1	3F	K	9
RT	302	E	GA-	1	3F	N	0
RT	305	E	GA-	1	3F	K	12
RT	305	E	GA-	1	3F	N	0
RT	316	G	GG-	1	3F, 3G	E	10
RT	316	G	GG-	1	3F, 3G	K	3
RT	316	G	GG-	1	3G	R	23
RT	324	D	GA-	1	3F	K	1
RT	337	W	TGG	2	3G	*	3
RT	344	E	GA-	1	3F	K	14
RT	344	E	GA-	1	3F	N	0
RT	352	G	GG-	1	3F, 3G	E	3
RT	352	G	GG-	1	3F, 3G	K	0
RT	352	G	GG-	1	3G	R	13
RT	352	G	GG-	1	3G	S	0
RT	364	D	GAT	1	3F	K	0
RT	378	E	GA-	1	3F	N	0
RT	383	W	TGG	2, 3	3F, 3G	*	3
RT	384	G	GG-	1	3F, 3G	E	2
RT	384	G	GG-	1	3F, 3G	K	0
RT	384	G	GG-	1	3G	R	2

RT	384	G	GG-	1	3G	S	0
RT	396	E	GA-	1	3F	K	8
RT	398	W	TGG	2, 3	3F, 3G	*	2
RT	401	W	TGG	2	3G	*	2
RT	402	W	TGG	2	3G	*	4
RT	404	E	GA-	1	3F	K	3
RT	406	W	TGG	2	3G	*	0
RT	410	W	TGG	2, 3	3F, 3G	*	3
RT	413	E	GA-	1	3F	K	3
RT	413	E	GA-	1	3F	N	0
RT	414	W	TGG	2, 3	3F, 3G	*	6
RT	415	E	GA-	1	3F	N	0
RT	426	W	TGG	2	3G	*	2
RT	430	E	GA-	1	3F	N	0
RT	436	G	GG-	1	3G	R	15
RT	436	G	GG-	1	3G	S	0
RT	438	E	GA-	1	3F	K	5
RT	443	D	GAT	1	3F	K	0
RT	443	D	GAT	1	3F	N	4
RT	444	G	GG-	1	3F, 3G	E	0
RT	444	G	GG-	1	3F, 3G	K	0
RT	444	G	GG-	1	3G	R	2
RT	444	G	GG-	1	3G	S	0
RT	449	E	GA-	1	3F	K	7
RT	453	G	GG-	1	3F, 3G	K	0
RT	453	G	GG-	1	3G	R	2
RT	453	G	GG-	1	3G	S	0
RT	456	G	GG-	1	3F, 3G	E	1
RT	456	G	GG-	1	3F, 3G	K	1
RT	456	G	GG-	1	3G	R	1
RT	456	G	GG-	1	3G	S	0
RT	462	G	GG-	1	3F, 3G	E	1
RT	462	G	GG-	1	3G	R	1
RT	462	G	GG-	1	3G	S	0
RT	471	D	GA-	1	3F	K	2
RT	478	E	GA-	1	3F	K	0
RT	478	E	GA-	1	3F	N	0
RT	488	D	GA-	1	3F	K	0
RT	488	D	GA-	1	3F	N	0
RT	490	G	GG-	1	3F, 3G	K	0
RT	490	G	GG-	1	3G	R	9
RT	498	D	GA-	1	3F	K	0
RT	498	D	GA-	1	3F	N	0
RT	504	G	GG-	1	3F, 3G	K	0
RT	504	G	GG-	1	3G	R	2
RT	504	G	GG-	1	3G	S	0
RT	511	D	GA-	1	3F	K	0
RT	514	E	GA-	1	3F	K	2
RT	516	E	GA-	1	3F	N	0

RT	523	E	GA-	1	3F	K	3
RT	523	E	GA-	1	3F	N	0
RT	535	W	TGG	2, 3	3F, 3G	*	4
RT	541	G	GG-	1	3F, 3G	E	1
RT	541	G	GG-	1	3F, 3G	K	1
RT	541	G	GG-	1	3G	R	1
RT	541	G	GG-	1	3G	S	0
RT	543	G	GG-	1	3F, 3G	E	1
RT	543	G	GG-	1	3F, 3G	K	0
RT	543	G	GG-	1	3G	R	3
RT	543	G	GG-	1	3G	S	0
RT	544	G	GG-	1	3F, 3G	E	1
RT	544	G	GG-	1	3F, 3G	K	0
RT	544	G	GG-	1	3G	R	2
RT	544	G	GG-	1	3G	S	0
RT	546	E	GA-	1	3F	N	0
RT	549	D	GA-	1	3F	K	0
RT	555	G	GG-	1	3F, 3G	E	3
RT	555	G	GG-	1	3F, 3G	K	0
RT	555	G	GG-	1	3G	R	3
RT	555	G	GG-	1	3G	S	0
IN	3	D	GAT	1	3F	K	0
IN	3	D	GAT	1	3F	N	13
IN	4	G	GG-	1	3F, 3G	K	3
IN	4	G	GG-	1	3G	R	5
IN	10	E	GA-	1	3F	K	3
IN	10	E	GA-	1	3F	N	0
IN	11	E	GA-	1	3F	K	0
IN	11	E	GA-	1	3F	N	0
IN	13	E	GA-	1	3F	K	3
IN	13	E	GA-	1	3F	N	0
IN	19	W	TGG	2, 3	3F, 3G	*	3
IN	25	D	GA-	1	3F	K	1
IN	25	D	GA-	1	3F	N	4
IN	35	E	GA-	1	3F	K	7
IN	35	E	GA-	1	3F	N	0
IN	41	D	GA-	1	3F	K	0
IN	47	G	GG-	1	3F, 3G	E	6
IN	47	G	GG-	1	3F, 3G	K	1
IN	47	G	GG-	1	3G	R	3
IN	47	G	GG-	1	3G	S	0
IN	48	E	GA-	1	3F	K	6
IN	48	E	GA-	1	3F	N	0
IN	52	G	GG-	1	3F, 3G	E	0
IN	52	G	GG-	1	3F, 3G	K	0
IN	52	G	GG-	1	3G	R	7
IN	52	G	GG-	1	3G	S	0
IN	55	D	GA-	1	3F	K	0
IN	59	G	GG-	1	3F, 3G	K	1

IN	59	G	GG-	1	3G	R	9
IN	59	G	GG-	1	3G	S	0
IN	61	W	TGG	2	3G	*	0
IN	64	D	GA-	1	3F	K	0
IN	64	D	GA-	1	3F	N	5
IN	69	E	GA-	1	3F	K	7
IN	69	E	GA-	1	3F	N	0
IN	70	G	GG-	1	3F, 3G	K	3
IN	70	G	GG-	1	3G	R	9
IN	70	G	GG-	1	3G	S	2
IN	82	G	GG-	1	3F, 3G	E	1
IN	82	G	GG-	1	3F, 3G	K	0
IN	82	G	GG-	1	3G	R	6
IN	82	G	GG-	1	3G	S	2
IN	85	E	GA-	1	3F	K	0
IN	85	E	GA-	1	3F	N	0
IN	87	E	GA-	1	3F	K	3
IN	87	E	GA-	1	3F	N	0
IN	92	E	GA-	1	3F	K	2
IN	94	G	GG-	1	3F, 3G	E	0
IN	94	G	GG-	1	3F, 3G	K	0
IN	94	G	GG-	1	3G	R	2
IN	94	G	GG-	1	3G	S	0
IN	96	E	GA-	1	3F	K	2
IN	106	G	GG-	1	3F, 3G	E	3
IN	106	G	GG-	1	3F, 3G	K	0
IN	106	G	GG-	1	3G	R	6
IN	108	W	TGG	2	3G	*	2
IN	116	D	GA-	1	3F	K	0
IN	116	D	GA-	1	3F	N	1
IN	118	G	GG-	1	3F, 3G	E	0
IN	118	G	GG-	1	3F, 3G	K	0
IN	118	G	GG-	1	3G	S	1
IN	131	W	TGG	2	3G	*	3
IN	132	W	TGG	2, 3	3F, 3G	*	3
IN	149	G	GG-	1	3F, 3G	E	3
IN	149	G	GG-	1	3F, 3G	K	0
IN	149	G	GG-	1	3G	R	3
IN	149	G	GG-	1	3G	S	1
IN	152	E	GA-	1	3F	K	13
IN	152	E	GA-	1	3F	N	0
IN	167	D	GA-	1	3F	K	1
IN	167	D	GA-	1	3F	N	2
IN	170	E	GA-	1	3F	K	5
IN	170	E	GA-	1	3F	N	0
IN	178	M	ATG	3	3G	I	1
IN	189	G	GGG	1, 2, 3	3F, 3G	E	1
IN	189	G	GGG	1, 2, 3	3F, 3G	K	0
IN	190	G	GGG	1, 2, 3	3F, 3G	E	1

IN	190	G	GGG	1, 2, 3	3F, 3G	K	0
IN	190	G	GGG	1, 3	3G	R	2
IN	192	G	GG-	1	3F, 3G	E	1
IN	192	G	GG-	1	3F, 3G	K	0
IN	192	G	GG-	1	3G	R	0
IN	192	G	GG-	1	3G	S	0
IN	197	G	GG-	1	3F, 3G	E	0
IN	197	G	GG-	1	3F, 3G	K	0
IN	197	G	GG-	1	3G	S	0
IN	198	E	GA-	1	3F	K	2
IN	198	E	GA-	1	3F	N	0
IN	202	D	GA-	1	3F	K	0
IN	202	D	GA-	1	3F	N	0
IN	207	D	GA-	1	3F	K	0
IN	212	E	GAA	1	3F	K	5
IN	224	R	CGG	2, 3	3F, 3G	Q	18
IN	229	D	GAC	1	3F	K	0
IN	232	D	GA-	1	3F	K	0
IN	235	W	TGG	2, 3	3F, 3G	*	5
IN	237	G	GGA	1, 2	3F	K	0
IN	237	G	GGA	1	3G	R	0
IN	243	W	TGG	2, 3	3F, 3G	*	2
IN	245	G	GGT	1	3F, 3G	E	0
IN	245	G	GGT	1	3F, 3G	K	0
IN	245	G	GGT	1	3G	S	0
IN	246	E	GAA	1	3F	K	3
IN	247	G	GG-	1	3F, 3G	E	0
IN	247	G	GG-	1	3F, 3G	K	1
IN	247	G	GG-	1	3G	R	4
IN	247	G	GG-	1	3G	S	0
IN	253	D	GA-	1	3F	K	0
IN	253	D	GA-	1	3F	N	3
IN	256	D	GA-	1	3F	K	1
IN	256	D	GA-	1	3F	N	0
IN	270	D	GA-	1	3F	K	0
IN	272	G	GGA	1, 2	3F	E	2
IN	272	G	GGA	1, 2	3F	K	1
IN	272	G	GGA	1	3G	R	9
IN	275	M	ATG	3	3G	I	0
IN	277	G	GG-	1	3F, 3G	E	0
IN	277	G	GG-	1	3F, 3G	K	0
IN	277	G	GG-	1	3G	S	1
IN	279	D	GAT	1	3F	K	0
IN	284	R	-GA	2	3F	Q	0
IN	287	E	GA-	1	3F	K	10
IN	287	E	GA-	1	3F	N	0

Supplementary Table 2. Estimated Proportion of APOBEC-Edited Sequences according to Number of Signature APOBEC-Associated Mutations

Number of Signature APOBEC-Associated Mutations	Proportion, %		
	protease	RT	Integrase
0	0	0	0
1	18	19	16
2	86	79	76
3	99.8	98.5	97.8
>=4	99.9	99.9	99.9

Local False Discovery Rate was used to estimate the rate for each number of APOBEC-associated mutations at which a sequence with that number of APOBEC-associated mutations did not arise from APOBEC-editing. The rates shown are the rates at which a sequence arose from APOBEC-editing.

Supplementary Table 3. Number of Sequences by Number of Very Rare Amino Acid Variants Occurring at a Prevalence <0.01%

No. Rare Variants	Protease		RT		Integrase	
	No. Sequences	%	No. Sequences	%	No. Sequences	%
0	106496	96.5	106497	89.7	11073	93.5
1	3338	3.0	9572	8.1	662	5.6
2	352	0.3	1571	1.3	66	0.6
3	95	0.1	501	0.4	11	0.1
4	50	0.0	211	0.2	14	0.1
5	31	0.0	103	0.1	5	0.0
6	13	0.0	68	0.1	2	0.0
7	12	0.0	36	0.0	1	0.0
8	11	0.0	29	0.0	2	0.0
9	3	0.0	24	0.0	2	0.0
>=10	6	0.0	71	0.1	2	0.0

The proportion of sequences from plasma samples.
The highest number of very rare amino acid variants in a sequence was 10, 29 and 15 for protease, RT and integrase, respectively.
